# Pancreatitis in A Patient with Cystic Fibrosis Taking Ivacaftor

**DOI:** 10.3390/children7010006

**Published:** 2020-01-12

**Authors:** Argyri Petrocheilou, Athanasios G. Kaditis, Ioanna Loukou

**Affiliations:** 1 Cystic fibrosis Department, Agia Sophia Children’s Hospital, 115 27 Athens, Greece; ioannaloukou@gmail.com; 2 Division of Pediatric Pulmonology, First Department of Pediatrics, National and Kapodistrian University of Athens School of Medicine and Aghia Sophia Children’s Hospital, 115 27 Athens, Greece; kaditia@hotmail.com

**Keywords:** pancreatitis, cystic fibrosis, ivacaftor, CFTR

## Abstract

Pancreatitis is rare in pancreatic insufficient cystic fibrosis patients. While pancreatic insufficiency has been considered irreversible until now, in the current era of new therapies with modulators of the Cystic Fibrosis Transmembrane Regulator CFTR channel, there are reports of improvement of pancreatic exocrine function. We describe the case of an adolescent with cystic fibrosis who developed pancreatitis after the partial recovery of pancreatic function while taking ivacaftor. This case adds to the limited body of evidence that CFTR modulators lead to the improvement of pancreatic exocrine function in cystic fibrosis.

## 1. Introduction

Pancreatitis in cystic fibrosis (CF) was first described in patients with cystic fibrosis who were pancreatic sufficient [1]. Pancreatitis has also been described in pancreatic insufficient patients, although that is very rare and, in many cases, the patients have only become insufficient after the pancreatitis episode [2].

Recently therapies in CF, such as Cystic Fibrosis Transmembrane Regulator (CFTR) potentiators, have been used in patients with specific genotypes with good results, resulting in at least partial recovery of CFTR function. In the recent report by Megalaa et al., a case of pancreatitis in a patient taking ivacaftor was described [3]. A second case of a CF patient with history of PI who developed pancreatitis while on treatment with ivacaftor is described below. This case adds to the body of evidence that at least partial restoration of pancreatic function is feasible with ivacaftor, and this is clinically significant.

## 2. Case

The adolescent and her parents were informed and parents consented for the publication of this case.

A 14-year old girl with CF was admitted to the hospital with a history of progressively increasing epigastric pain and nausea. She was otherwise well, reporting only mild cough secondary to recent respiratory infection that was improving. The patient’s medical history was remarkable for CF diagnosis with the genotype F508del/G551D. Patient’s oral medications included ivacaftor 150mg twice daily that was started four years prior to hospital admission. The adolescent had almost discontinued pancreatic enzyme replacement therapy (PERT) and was using PERT only with very fatty meals since about 2 years after starting ivacaftor as PI symptoms were absent and BMI had increased from 21 to 27 kg/m^2^. The patient’s diet was rich in meals with a high fat content, such as fast food restaurant meals and fried food items.

The patient was afebrile and physical examination of the chest revealed few crackles at the lung bases while the abdomen was diffusely tender on palpation and bowel sounds were present. Further, no hepatosplenomegaly was noted. The laboratory values on admission were notable for an amylase of 197 U/L (normal range: 28-100U/L) and a lipase of 647 (normal range: <190 U/L). Thus, the diagnosis of pancreatitis was entertained based on the history of epigastric pain, findings from examination of the abdomen and laboratory results.

The adolescent was treated with intravenous fluids and was discharged in good condition after four days later. No recurrence of pancreatitis symptoms has been noted up to more than two years after the episode. Ivacaftor was restarted shortly after discharge and was well tolerated. Stool elastase was measured almost two years after the episode and was 159 μgE/g, indicating moderate pancreatic insufficiency (normal >200 μgE/g, moderate pancreatic insufficiency 100–200 μgE/g, severe pancreatic insufficiency <100 μgE/g). Stool elastase was not measured on diagnosis or before starting ivacaftor. The patient to date denies PI symptoms and continues to use PERT only with very fatty meals.

## 3. Discussion

This case further underlines the importance of the point made by Megalaa et al. that clinicians should consider measuring stool elastase on patients taking ivacaftor to determine if pancreatic function has recovered [3], especially in light of safety and efficacy studies of ivacafor, (KIWI and ARRIVAL) results [4,5] which also suggest that ivacaftor leads to recovery of pancreatic function. Unlike the patient described by Megalaa et al. that had a normal stool elastase, over 500 mcg/g right after the pancreatitis episode and 376 mcg/g shortly after the pancreatitis episode, [3] the patient described here had a stool elastase that was in the moderate pancreatic insufficiency range.

Stool elastase in our patient was measured much later than the pancreatitis episode, but based on the clinical symptoms it can be presumed that stool elastase value was similar at the time of the pancreatitis as well. Despite moderate pancreatic insufficiency, the patient still experienced an acute pancreatitis episode that led to hospitalization. Therefore, pancreatitis in this PI patient could be explained by the improvement of CFTR function with ivacaftor, by a similar mechanism to the one proposed by Carrion et al. for the reduction of pancreatitis frequency and recurrence risk [6].

In the current era of CFTR modulators, the question raised by the CF community, especially by patients and parents, concerns whether CFTR modulators will reduce the treatment burden. Consequently, data suggesting the restoration of pancreatic function that could lead to discontinuation of PERT are particularly important. Furthermore, pancreatitis is generally uncommon in PI patients, so the description of pancreatitis in previously PI patients on ivacaftor implies at least partial restoration of pancreatic function. Therefore, clinicians and patients should be alert to development of pancreatitis symptoms in patients treated with ivacaftor. Patient and parent education preemptively, during clinic visits, should be considered in order to prevent delays in appropriate treatment.
